# Hypofractionation Adoption in Prostate Cancer Radiotherapy: Results of an International Survey

**DOI:** 10.1200/GO.23.00046

**Published:** 2023-06-15

**Authors:** Ali Sabbagh, Jessica Weiss, Bouchra Tawk, Mohammed A. Mohammed, Hasan Abdulbaki, Fabio Y. Moraes, Surbhi Grover, Mei Ling Yap, Eduardo Zubizarreta, Yolande Lievens, Danielle Rodin, Osama Mohamad

**Affiliations:** ^1^ Department of Radiation Oncology, University of California San Francisco, San Francisco, CA; ^2^ American University of Beirut Medical Center, Beirut, Lebanon; ^3^ Department of Biostatistics, Princess Margaret Cancer Centre, University of Toronto, Toronto, ON, Canada; ^4^ Clinical Cooperation Unit—Translational Radiation Oncology, National Center for Tumoral Diseases NCT Heidelberg, German Cancer Research Center (DKFZ), Heidelberg University Hospital, Heidelberg, Germany; ^5^ Department of Radiation Oncology, University of Pittsburgh Medical Center, Pittsburgh, PA; ^6^ Department of Oncology, Division of Radiation Oncology, Queen's University, Kingston, ON, Canada; ^7^ Department of Radiation Oncology, University of Pennsylvania, Philadelphia, Philadelphia, PA; ^8^ Perelman School of Medicine, University of Pennsylvania, Philadelphia, PA; ^9^ Collaboration for Cancer Outcomes, Research and Evaluation (CCORE), Ingham Institute, UNSW Sydney, Liverpool, NSW, Australia; ^10^ Liverpool and Macarthur Cancer Therapy Centres, Western Sydney University, Campbelltown, NSW, Australia; ^11^ The George Institute for Global Health, UNSW Sydney, Newtown, NSW, Australia; ^12^ International Atomic Energy Agency, Vienna, Austria; ^13^ Ghent University Hospital and Ghent University, Ghent, Belgium; ^14^ Radiation Medicine Program, Princess Margaret Cancer Centre, Toronto, ON, Canada; ^15^ Department of Radiation Oncology, University of Toronto, ON, Canada; ^16^ Department of Urology, University of California San Francisco, San Francisco, CA

## Abstract

**PURPOSE:**

Hypofractionation is noninferior to conventional fractionation in the treatment of localized prostate cancer. Using results from the European Society of Radiation Oncology's (ESTRO) Global Impact of Radiotherapy in Oncology (GIRO) initiative survey on hypofractionation, this study identifies rates of adoption, facilitating factors, and barriers to adoption of hypofractionation in prostate cancer across World Bank income groups.

**MATERIALS AND METHODS:**

The ESTRO-GIRO initiative administered an international, anonymous, electronic survey to radiation oncologists from 2018 to 2019. Physician demographics, clinical practice characteristics, and hypofractionation regimen use (if any) for several prostate cancer scenarios were collected. Responders were asked about specific justifications and barriers to adopting hypofractionation, and responses were stratified by World Bank income group. Multivariate logistic regression models were used to analyze variables associated with hypofractionation preference.

**RESULTS:**

A total of 1,157 physician responses were included. Most respondents (60%) were from high-income countries (HICs). In the curative setting, hypofractionation was most often preferred in low- and intermediate-risk prostate cancers, with 52% and 47% of respondents reporting hypofractionation use in ≥50% of patients, respectively. These rates drop to 35% and 20% in high-risk prostate cancer and where pelvic irradiation is indicated. Most respondents (89%) preferred hypofractionation in the palliative setting. Overall, respondents from upper-middle-income countries and lower-middle- and low-income countries were significantly less likely to prefer hypofractionation than those from HICs (*P* < .001). The most frequently cited justification and barrier were availability of published evidence and fear of worse late toxicity, respectively.

**CONCLUSION:**

Hypofractionation preference varies by indication and World Bank income group, with greater acceptance among providers in HICs for all indications. These results provide a basis for targeted interventions to increase provider acceptance of this treatment modality.

## INTRODUCTION

Prostate cancer is the second most commonly diagnosed cancer and the fifth leading cause of death in men worldwide.^1^ Radiation therapy is one of the main modalities of prostate cancer treatment, capable of curing patients in appropriate clinical settings.^2^ Despite these benefits, the supply of radiotherapy centers worldwide is insufficient to keep up with demand, with over 90% of low-income countries lacking adequate access.^3^ The use of hypofractionation, that is, delivering larger doses of radiation over shorter periods of time, can improve access to care and decrease the cost of treatment. Several large randomized trials have consistently shown that a hypofractionated radiation therapy regimen is noninferior to conventionally fractionated treatments in low- and intermediate-risk prostate cancer, with some evidence also supporting its use in high-risk disease.^4-8^ More recently, the ultra-hypofractionated versus conventionally fractionated radiotherapy for prostate cancer (HYPO-RT-PC) and intensity-modulated radiotherapy versus stereotactic body radiotherapy for prostate cancer (PACE-B) trials have shown that ultra-hypofractionation is another feasible alternative to conventional radiation regimens, particularly for patients with intermediate-risk disease, offering even further convenience to patients and improved access to care.^9,10^ Nonetheless, the factors influencing hypofractionation uptake and use in low-resource settings are not clearly understood.^2^

**CONTEXT**

**Key Objective**
When do radiation oncologists prefer hypofractionation in treating prostate cancer and what are the factors that encourage or discourage its use in different resource settings?
**Knowledge Generated**
Although hypofractionation is widely accepted for palliative treatment, its use for other indications varies considerably. Across all indications, radiation oncologists from high-income countries have been found to consistently prefer hypofractionation over their peers from middle- and low-income countries. Adoption was mainly favored by the availability of published evidence, with worse late toxicity cited as a major concern.
**Relevance**
Interventions that target barriers to adoption of hypofractionation are vital in improving patient access to adequate care.

The data on the rate of adoption of hypofractionation for prostate cancer is very limited. In 2020, the European Society of Radiotherapy and Oncology's Global Impact of Radiotherapy in Oncology (ESTRO-GIRO) initiative released a survey analyzing global trends of hypofractionation use in clinical practice across several disease sites.^11^ The results showed significant variability in hypofractionation utilization across different indications and geographic locations. However, trends across income groups and disease sites were not reported. An understanding of this difference is crucial to promote the adoption of emerging evidence into everyday clinical practice. This study will use the results from the ESTRO-GIRO survey to illustrate the preference of hypofractionation for prostate cancer across World Bank income groups, while discussing factors that enable and limit its adoption.

## MATERIALS AND METHODS

The ESTRO-GIRO initiative survey was distributed to radiation oncologists between January 2018 and January 2019 through the ESTRO membership database and liaisons of several national and regional professional radiation oncology societies globally. The survey was filled electronically using an online platform, and responses were anonymous. This study was exempt from institutional review board review. Details on survey design have been described previously.^11,12^ Respondents provided information on their demographics, clinical expertise, and available department resources. Full survey questions and other details are presented in the Data Supplement.

Those who reported treating at least one case of prostate cancer per month were asked follow-up questions on their patterns of practice. They were presented with five different clinical scenarios and asked to specify their preference for hypofractionation (>2 Gy per fraction), conventional fractionation (≤2 Gy per fraction), or both. Respondents were classified as hypofractionation users if they reported using hypofractionation or hypofractionation and conventional fractionation with a preference for hypofractionation in ≥50% of their patients and in ≥50% of clinical scenarios. Respondents who chose both were asked for the proportion of patients they opted to treat with hypofractionation. Those who chose hypofractionation were asked for the dose per fraction and the number of fractions used. Clinical scenarios included were as follows: (1) low-risk disease, (2) intermediate-risk disease, (3) high-risk disease, (4) pelvic irradiation, and (5) palliative symptom control. Respondents had to subsequently select the justifications or barriers that influenced their choice of treatment, with 11 different justifications and nine different barriers listed as options.

Counts and percentages were reported for descriptive statistics. Comparisons were performed using the *t* test for continuous variables and the chi-square or Fisher's exact test for categorical variables. World Bank income groups included high-income countries (HICs), upper-middle-income countries (UMICs), and lower middle- and low-income countries (LLMIC). The association between hypofractionation use and baseline characteristics of respondents was assessed using multivariate logistic regression. The following covariates were included in the model: sex, age, years of practice, region of current practice, World Bank income group, university affiliation, size of patient catchment area, and available technology. Associations from models are reported as odds ratios with 95% CIs, and Fisher's exact or chi-square test was used to analyze the association of justifications and barriers with the income group. A *P* value of <.05 was considered statistically significant, and all statistical tests were two-sided. All analyses were conducted using the R statistical environment, version 3.5.2 (R Foundation for Statistical Computing, Vienna, Austria).

## RESULTS

A total of 2,316 radiation oncologists completed the general survey, of whom 2,170 respondents treated at least one prostate cancer case per month. Of those respondents who managed prostate cancer, 1,013 did not respond to at least one of the prostate cancer scenarios, leading to an eventual sample size of 1,157. Because of the way the survey was distributed, we could not obtain an overall survey response rate. Respondent characteristics are shown in Table 1. Most respondents (698, 60%) were from HICs, 307 (27%) from UMICs, and 152 (13%) from LLMICs. Most respondents (813 or 70%) have been in practice for more than 5 years, with the majority (51%) being younger than 45 years and male (63%). Ninety-five percent of respondents report access to linear accelerators, and most reported access to advanced radiotherapy planning techniques, namely, intensity-modulated radiotherapy (IMRT) (88%), three-dimensional (3D) conformal radiotherapy (92%), and computed tomography (CT)–based planning (92%).

**TABLE 1:**
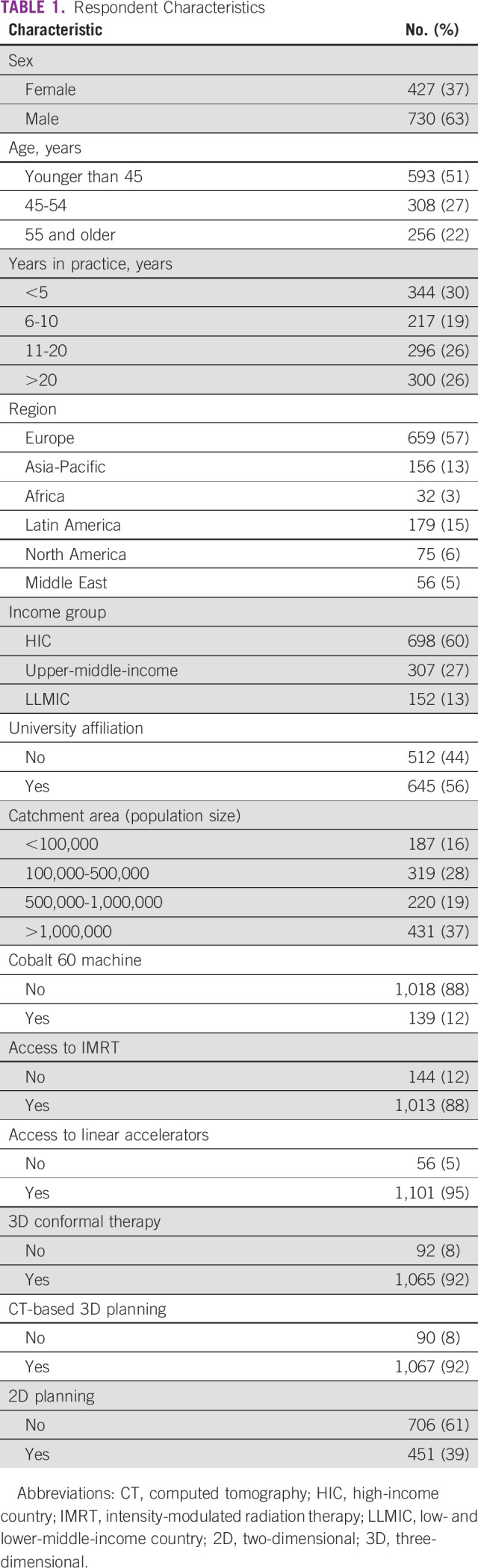
Respondent Characteristics

**FIG 1:**
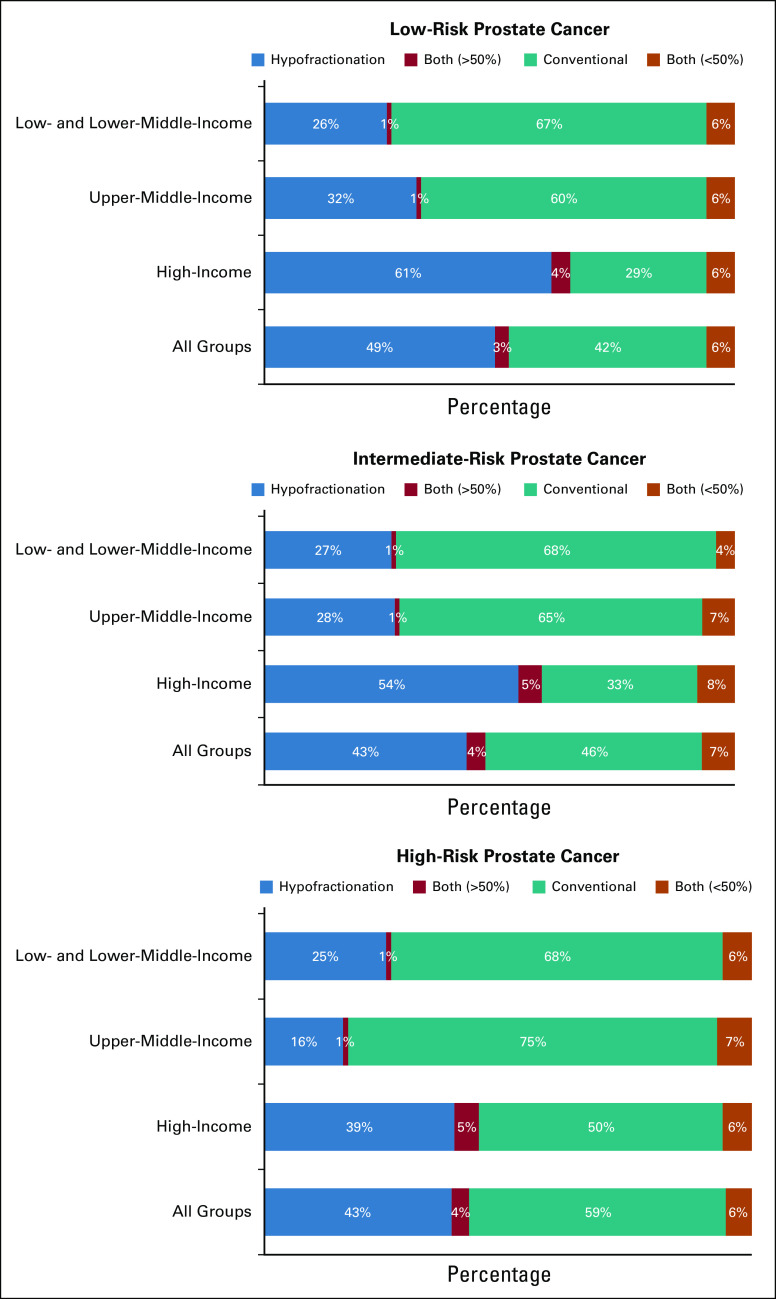

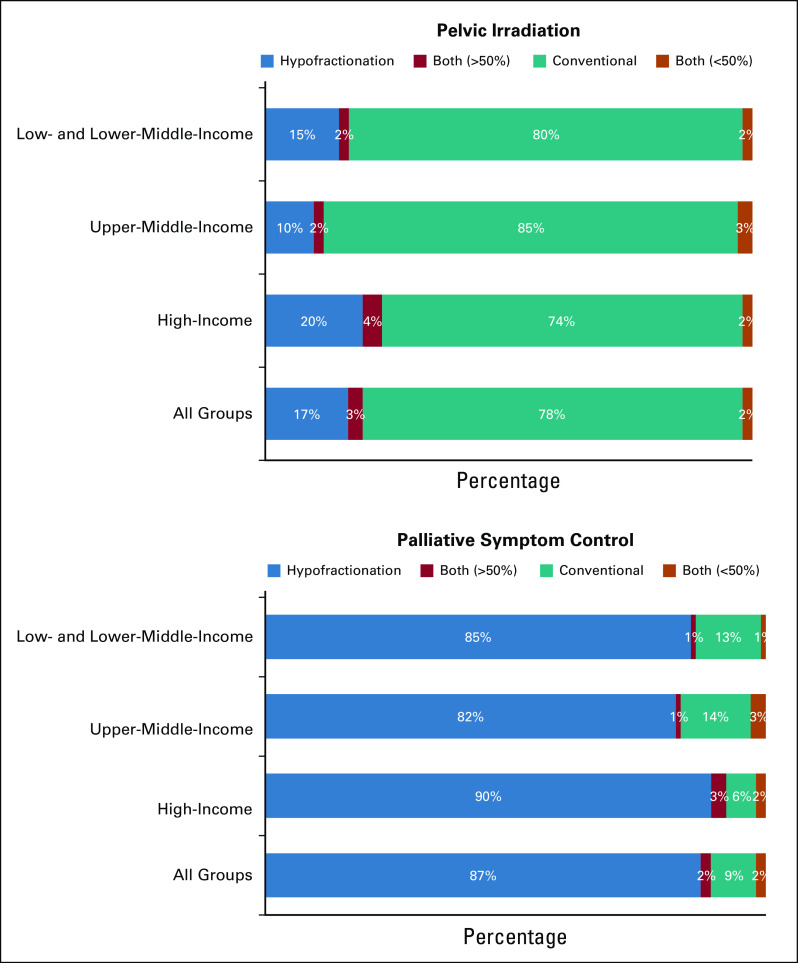
Utilization of hypofractionation for each clinical scenario by World Bank income group region. Both (>50%) refer to respondents who chose both hypofractionation and conventional fractionation for a particular scenario but with preference for hypofractionation in >50% of the cases. Both (<50%) refer to respondents who chose both hypofractionation and conventional fractionation for a particular scenario but with preference for hypofractionation in <50% of the cases.

Utilization of hypofractionation in each clinical scenario, stratified by income group region, is shown in Figure 1 and the Data Supplement. In the curative setting, low-risk prostate cancer had the highest rate of use of hypofractionation, with 52% of overall respondents reporting it as their preferred modality or as the modality used in >50% of their patients (Fig 1A). Similar rates were observed for intermediate-risk disease, where hypofractionation is preferred or used in >50% of patients by 59%, 29%, and 28% of practitioners from HICs, UMICs, and LLMICs, respectively (Fig 1B). Rates of adoption become noticeably lower in high-risk prostate cancer and in cases of pelvic irradiation, with only 35% and 20% of overall respondents reporting a preference for the modality or use in >50% of patients in these scenarios, respectively (Figs 1C and 1D). In the palliative setting, however, hypofractionation is highly used across income groups (87% overall, 90% in HICs, 82% in UMICs, and 85% in LLMICs; Fig 1E).

**FIG 2:**
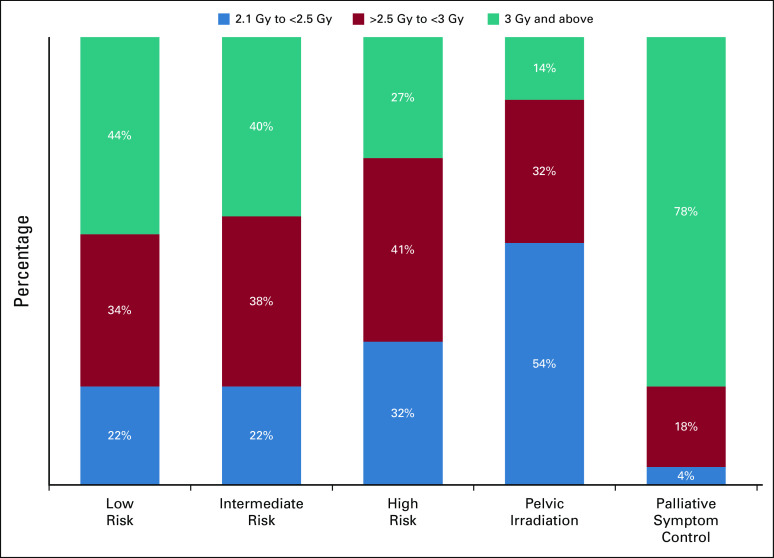
Preferred hypofractionation dose per fraction per scenario (in percentage).

Respondents who chose hypofractionation were asked about the dose and fractionation regimen that they used (Fig 2; Data Supplement [eTable 3]). For scenarios 1-3, most respondents used doses between 2.5 and 3.5 Gy. In the case of pelvic irradiation, 54% of respondents used doses of 2.1 Gy to <2.5 Gy, with 32% using 2.5 Gy to <3 Gy. Doses >5 Gy were used in cases of low- and intermediate-risk prostate cancers in the curative setting (9% and 5%, respectively) and for palliative symptom control (12%) but rarely for other scenarios.

Multivariate logistic regression for the association between hypofractionation use and baseline characteristics of respondents is shown in Table 2. Relative to HICs, respondents from UMICs (odds ratio [OR], 0.27; 95% CI, 0.18 to 0.40) were less likely to use hypofractionation, with similar results for respondents from LLMICs (OR, 0.40; 95% CI, 0.22 to 0.72). The geographic region of respondents was not significantly associated with hypofractionation (HF) use (*P* = .13). The association between the region and the use of hypofractionation is noticeably different when stratified by income group (Data Supplement [eTable 4]). In HICs, respondents from the Middle East, Latin America, or Asia-Pacific are less likely to use hypofractionation than their peers from Europe, with respondents from North America being more likely to use hypofractionation. In UMICs and LLMICs, the association between the region and the use of hypofractionation remains nonsignificant (*P* = .76 and *P* = .98, respectively). Overall, respondents practicing in catchment areas with a population of >1 million were 82% more likely to use hypofractionation relative to those practicing in catchment areas with populations of <100,000. When stratified on the basis of the income group, this association persists in HICs (OR, 2.43; 95% CI, 1.38 to 4.29; *P* = .0022), but is no longer significant in UMICs and LLMICs (*P* = .54 and *P* = .39, respectively). Respondents who use IMRT were greater than four times more likely to use hypofractionation, with the association maintained in a statistically significant manner across all income groups. Age, sex, years of practice, university affiliation, having Cobalt-60 machines, access to linear accelerators, 3D conformal radiotherapy, and CT-based 3D planning had no significant impact on the likelihood of hypofractionation overall and within specific income groups.

**TABLE 2:**
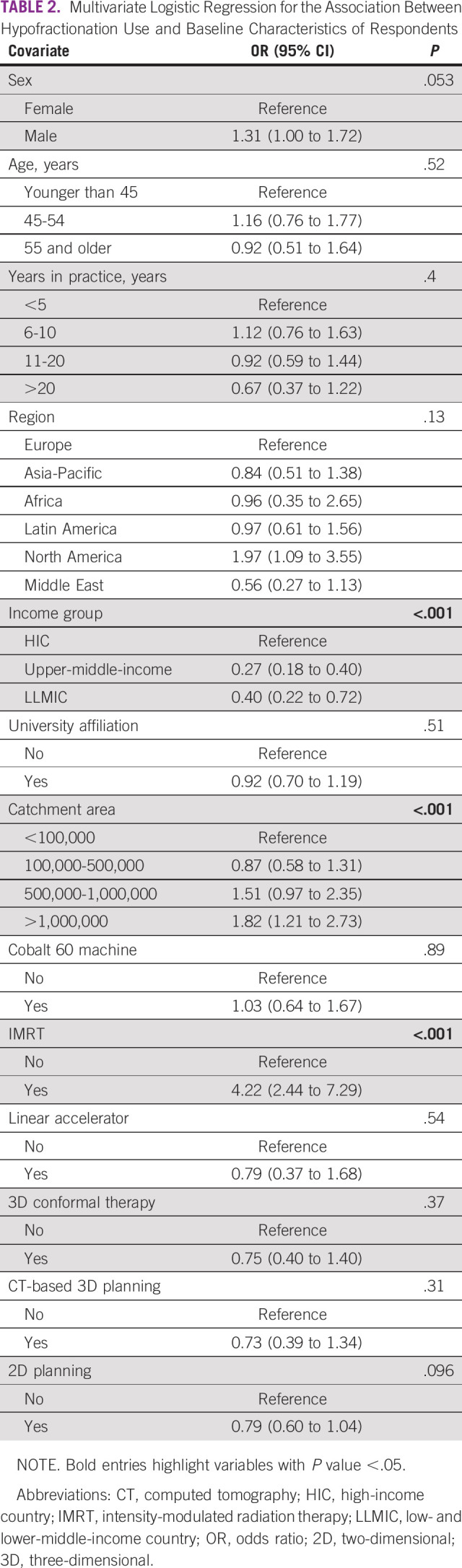
Multivariate Logistic Regression for the Association Between Hypofractionation Use and Baseline Characteristics of Respondents

Responses concerning justifications and barriers that influence physicians' use of hypofractionation were also stratified according to the World Bank income group (Table 3). The most frequently cited justification overall was the presence of published evidence supporting hypofractionation (75%). Respondents from HICs (79%) cited this justification more frequently compared with respondents from UMICs (70%) and LLMICs (66%; *P* < .001). Similarly, equivalent local control and toxicity profile were cited by 66% and 52% of respondents, respectively, as justifications for the use of hypofractionation, with respondents from HICs also being more likely to cite these causes. Twenty-nine percent of respondents cited previous clinical experience, and only 4% cited better reimbursement as justifications, with no significant differences between income groups (*P* = .068 and *P* = .13, respectively). Patient preference (38% in HICs *v* 19% in UMICs and 15% in LLMICs; *P* < .001) and the influence of peer practice (35% in HICs *v* 16% in UMICs and 21% in LLMICs; *P* < .001) were more frequently reported by respondents from HICs than those in UMICs and LLMICs. Fifty-four percent of practitioners reported patient convenience as justification (61% in HICs, 42% in UMICs, 43% in LLMICs; *P* < .001). Respondents from HICs were also more likely to cite efficient use of resources and a personal preference for elderly patients with multiple comorbidities as justifications for hypofractionation compared with their peers from UMICs and LLMICs, with a statistically significant difference between income groups (*P* < .001). Fear of worse late toxicity was the most cited barrier overall (40%). This is followed by fear of worse acute toxicity (29%) and lack of sufficient long-term data (28%). There was no statistically significant difference between World Bank income groups (*P* > .05) regarding these barriers. Other barriers were less frequently reported.

**TABLE 3:**
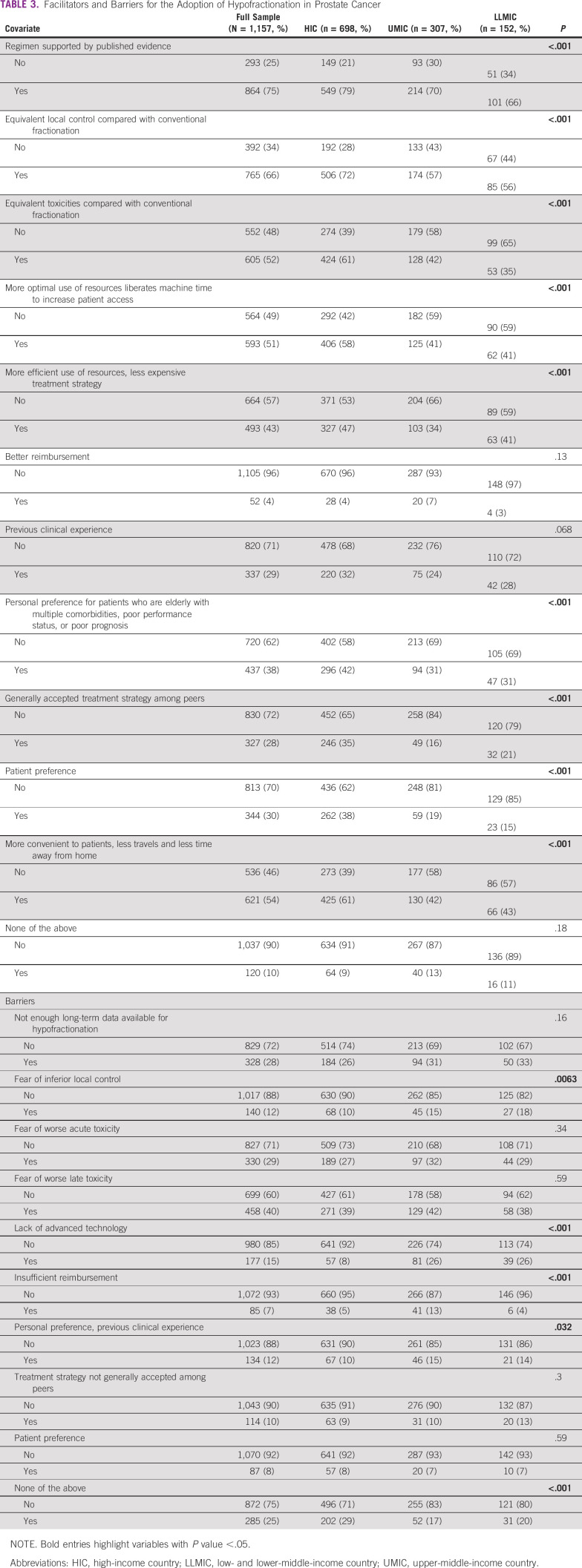
Facilitators and Barriers for the Adoption of Hypofractionation in Prostate Cancer

## DISCUSSION

The results of this international survey on the use of hypofractionation in prostate cancer radiotherapy show heterogeneous patterns of uptake across curative indications and income levels, with respondents from UMICs and LLMICs reporting significantly lower uptake than respondents from HICs. Respondents preferred using hypofractionation in low- and intermediate-risk prostate cancers, likely because of the abundance of published evidence supporting its use in these scenarios.^4,7,8,10^ Conversely, high-risk prostate cancer and cases of pelvic irradiation were less likely to be treated with hypofractionation. Although clinical trials at the time included patients with high-risk disease, they constituted only a minority of the studies' cohorts. Providers might have therefore preferred to exercise caution in applying trial results to patients with high-risk disease. In 2018, American Society for Therapeutic Radiology and Oncology (ASTRO), ASCO, and American Urological Association (AUA) published joint guidelines recommending hypofractionation for the treatment of localized high-risk prostate cancer,^13^ specifically stating that high-risk patients were reasonably well represented in the completed trials. We therefore expect these new guidelines to address provider hesitation concerning the availability of evidence and lead to increased adoption of hypofractionation for the treatment of high-risk disease.

Most importantly, despite published evidence being the most frequently cited justification for the use of hypofractionation in all income groups and the plethora of published evidence on the noninferiority of hypofractionation in low- and intermediate-risk diseases, income group remained significantly associated with adopting hypofractionation in these scenarios. Although it might be argued that these differences can be due to the lack of access to proper technology, our results were adjusted for access to IMRT and other technologies. This is not to say, however, that lack of access is not an independent barrier to adoption. In fact, providers in UMICs and LLMICs were significantly more likely to cite lack of advanced technology as a barrier to the use of hypofractionation. Furthermore, the gap in access to treatment equipment is striking, with LLMICs having access to <10% of global teletherapy machines, despite >70% of the annual incidence of cancer occurring in these countries.^14^ Hypofractionation therefore offers an important advantage as it allows for more efficient use of available resources. Another explanation for this discrepancy could be that patients from UMICs and LLMICs are under-represented in clinical trials. Current published evidence is derived from trials conducted almost exclusively on patients from HICs including landmark trials such as HYPO-RT-PC, prostate fractionated irradiation trial (PROFIT), PACE-B, and Radiation Therapy Oncology Group 0415.^7-10^ This underlines the need for studies that recruit patients from all backgrounds to facilitate the adoption of hypofractionation in regions where a treatment modality is underused. Other possible reasons include delayed access to literature, especially as access may be limited by cost.^15^

Although physicians in HICs were consistently more likely to use hypofractionation than respondents from UMICs and LLMICs across most clinical scenarios, respondents from LLMICs were 13% more likely to prefer hypofractionation for pelvic irradiation. This is an interesting finding as most published guidelines supporting the use of hypofractionation did not include trials involving pelvic lymph node treatment,^13^ which would fall under this category. As these guidelines were released toward the end of our survey, it might be that this association has changed. Nonetheless, it could reflect an underlying knowledge gap and future studies should investigate this finding more closely. Interestingly, respondents from HICs cited patient preference as a justification for the use of hypofractionation at nearly twice the rate of respondents from LLMICs and UMICs. This is probably due to the emphasis placed on shared decision making in HICs, which has proven to be beneficial in the setting of cancer care,^16,17^ but is not as widely adopted in developing countries. This parallels our previous findings analyzing preferences for hypofractionation in breast cancer.^12^

A recent US population–based analysis found that adopting moderate hypofractionation for localized prostate cancer could lead to annual savings between 25% and 50%, which has the potential to affect provider and departmental revenues.^18^ This raises concern that departments and providers might push for conventional fractionation to avoid an impact on revenue. Nonetheless, only 7% of respondents reported insufficient reimbursement as a barrier to adopting hypofractionation and 51% cited efficient use of resources as a justification for their use of hypofractionation. Although encouraging, it is important to keep in mind that these numbers are self-reported, and therefore, systemic policies should be set in place to prevent such practices.

A minority of physicians used ultra-hypofractionation in nonpalliative settings (<10%). It should be noted, however, that this survey was distributed before the results of HYPO-RT-PC were available. HYPO-RT-PC showed that ultra-hypofractionated radiotherapy is noninferior to conventionally fractionated regimens in localized prostate cancer in terms of failure-free survival.^9^ This has important implications on cost, accessibility, and patient convenience, because of the considerable reduction in treatment duration associated with ultra-hypofractionation. In fact, for the majority of National Comprehensive Cancer Network risk groups, current guidelines approve ultra-hypofractionation as an appropriate treatment regimen and the entire dose of radiation can be administered in ≤7 fractions versus ≥20 fractions with moderate hypofractionation,^19^ decreasing required treatment time by weeks. In the wake of the COVID-19 pandemic, the results of HYPO-RT-PC paved the way for the recommendation of ultra-hypofractionation as a means of minimizing the risk of exposure in unfavorable-risk patients.^20,21^

Our discussion would not be complete without addressing the study’s limitations. As was just discussed, this survey was distributed before the results of HYPO-RT-PC and PACE-B, the joint ASTRO, ASCO, and AUA guidelines, and the COVID-19 pandemic. A change in treatment guidelines would likely have led to an increase in the rate of uptake of moderate and ultra-hypofractionation, especially in the setting of the global pandemic. Furthermore, the survey was shared through professional society membership databases, which can lead to the possibility of selection bias and affect the accuracy and generalizability of our results. Nonetheless, our results shed light on factors that have the highest impact on provider acceptance of hypofractionation, particularly in UMIC and LLMICs. This makes them crucial for more efficient design of clinical trials, policies, and administrative decisions that would increase the use of HF in eligible patients and decrease the financial and logistical burdens in low-resource settings.

In conclusion, the results of this study show that hypofractionation adoption differs significantly across World Bank income groups and indications. Despite available evidence supporting the use of the treatment modality in several clinical scenarios, several barriers still exist that prevent its utilization, with fear of worse late toxicity being the most frequently cited factor. Interventions targeting these factors have the potential to increase hypofractionation adoption, particularly in UMICs and LLMICs.
